# 

**Published:** 1907-03

**Authors:** 


### THE BRISTOL

### Medico-Chirurgical Journal.

A JOURNAL OF THE MEDICAL SCIENCES FOR THE WEST OF ENGLAND AND SOUTH WALES

*PUBLISHED UNDER THE AUSPICES OF*

*THE BRISTOL MEDICO-CHIRURGICAL SOCIETY.*

EDITOR:

R. SHINGLETON SMITH, M.D., B.Sc.,

WITH WHOM ARE ASSOCIATED

J. MICHELL CLARKE, M.A., M.D., J. LACY FIRTH, M.S., M.D., AND JAMES SWAIN, M.S., M.D

ASSISTANT-EDITOR:

P. WATSON WILLIAMS, M.D.

EDITORIAL SECRETARY:

JAMES TAYLOR

"Scire est nescire, nisi id me Scire alius sciret."

LIBRARY

SURGEON GENERAL'S OFFICE

SEP-28-1908

199723.

VOL. XXV.

BRISTOL: J. W. ARROWSMITH.

LONDON: J. & A. CHURCHILL, 7 GREAT MARLBOROUGH STREET.

1907.

WI
BR323

J.W. Arrowsmith Publisher Bristol

### CONTENTS OF VOLUME XXV.

*Page*

Prognosis. By P. H. PYE-SMITH, M.D., F.R.C.P., F.R.S. .. .. 1

Cerebro-Spinal Fever (Epidemic Cerebro-Spinal Meningitis). By D. S. DAVIES, M.D. Lond., and I. WALKER HALL, M.D. Vict... 14

Operations for Deflections and Spurs of the Nasal Septum, with special reference to Sub-mucous Resection. By PATRICK WATSON WILLIAMS, M.D. Lond. .. .. .. .. 21

Two Cases of Ruptured Intestine. By HAROLD F. MOLE, F.R.C.S. 38

Concluding Notes on a Case of Splenomegalic Cirrhosis in a Child aged seven years. By EDWARD CECIL WILLIAMS, M.B.Cantab. With post-mortem notes by J. M. FORTESCUE-BRICKDALE, M.A., M.D. Oxon. .. .. .. .. .. .. .. 43

Acquired Angioma of the Liver. By A. LEWIN SHEPPARD, M.B., B.S. Durh. .. .. .. 46

The Sanatorium Treatment of Pulmonary Tuberculosis—Is it a Success? By D. J. CHOWRY MUTHU, M.D. .. .. .. 50

Some Notes on Styracol. By CHARLES REINHARDT, M.D... .. 54

Obituary Notice—
EDWARD MARKHAM SKERRITT, M.D. Lond., F.R.C.P. .. 97

Cases Illustrating the More Unusual Complications of Pneumonia. By J. MICHELL CLARKE, M.A., M.D. Cantab., F.R.C.P. .. 108

The Value of Compression of the Aorta in the Treatment of Post-Partum Hemorrhage. By F. PERCY ELLIOTT, M.B. Aberd. 121, 310

Eighty Cases of Lupus Vulgaris. By W. KENNETH WILLS, M.D. Cantab. .. .. .. 127

A Case of Narcolepsy. By BERTRAM M. H. ROGERS, M.D. Oxon... 144

Tumours and Tubercle in Monkeys. By W. ROGER WILLIAMS, F.R.C.S. .. .. .. .. 149

Rheumatic Carditis in Childhood. By CAREY COOMBS, M.D. Lond. 193

Treatment of Graves's Disease by Anti-Thyreoid Serum and by X-Rays. By J. MICHELL CLARKE, M.A., M.D.Cantab., F.R.C.P. 201

CONTENTS.

| | Page |
|---|---|
| Cerebral Lesions in Pregnancy and Parturition. By WALTER C. SWAYNE, M.D., B.S. Lond. .. .. .. .. .. | 209 |
| A Suggested Treatment for Functional Aphonia. By C. PERCIVAL CROUCH, M.B. Lond., F.R.C.S. .. .. .. .. .. | 214 |
| A Case of Filariasis: Removal of Lymphatic Varix by Operation. By J. PAUL BUSH, C.M.G., and J. A. NIXON, M.B. Cantab., M.R.C.P. .. .. .. .. .. .. .. .. | 218 |
| The Medical Reading Society, Bristol. By L. M. GRIFFITHS .. | 222 |
| Syphilis. By HENRY WALDO, M.D... .. .. .. .. | 289 |
| Some Remarks on Spinal Anæsthesia as based upon the Personal Observation of Thirty Cases. By ERNEST W. HEY GROVES, M.S. Lond., F.R.C.S. .. .. .. .. .. .. | 305 |
| The Value of Compression of the Aorta in the Treatment of Post-Partum Hemorrhage (*continued*). By F. PERCY ELLIOTT, M.B. | 310 |
| A Case of Generalised Sarcoma, with Blood Changes. By F. G. BUSHNELL, M.D. .. .. .. .. .. .. .. | 321 |
| Proportional Representation and the Comparison of Radiographs. By WILLIAM COTTON, M.A., M.D., D.P.H. .. .. .. | 326 |
| Public Health in Bristol. 1906–7. By D. S. DAVIES, M.D... .. | 336 |
| PROGRESS OF THE MEDICAL SCIENCES .. 57, 152, 236, | 341 |
| REVIEWS OF BOOKS .. .. .. .. 69, 168, 251, | 353 |
| EDITORIAL NOTES .. .. .. .. 76, 177, 272, | 368 |
| NOTES ON PREPARATIONS FOR THE SICK .. 83, 180, 278, | 374 |
| THE LIBRARY OF THE BRISTOL MEDICO-CHIRURGICAL SOCIETY .. .. .. .. 85, 182, 283, | 379 |
| MEETINGS OF SOCIETIES .. .. .. 87, 185, 285, | 382 |
| OBITUARY NOTICES .. .. .. .. .. .. 90, | 97 |
| LOCAL MEDICAL NOTES .. .. .. 93, 187, 287, | 383 |

**Soloid—Eosin-azur.**—BURROUGHS, WELLCOME & Co., London. —We have examined this "Soloid." Each soloid contains 0.015 gm. of Giemsa's stain, and is dissolved in 5 c.c. of pure methyl alcohol. When the staining directions are properly followed, the results are thoroughly satisfactory. These "soloid" stains are most convenient for use, and are reliable as regards their staining properties.

### The Library of the Bristol Medico-Chirurgical Society.

*The following donations have been received since the publication of the List in December:*

*February 28th,* 1907.

| | |
|---|---|
| J. Paul Bush, C.M.G. (1) .. .. .. .. | 5 volumes. |
| Medical Officer of the London County Council (2) .. | 1 volume. |
| Middlesex Hospital (3) .. .. .. .. | 1 ,, |
| The Pathological Society of London (4) .. .. | 2 volumes. |
| The Council of the Royal College of Physicians of London (5) .. .. .. .. .. | 1 volume. |
| Scholastic Trading Company (6) .. .. .. | 1 ,, |
| James Swain, M.D. (7) .. .. .. .. | 1 ,, |
| The Director of the U.S. Census Report (per W. Roger Williams) (8) .. .. .. .. .. | 2 volumes. |

Unbound periodicals have been received from Mr. Paul Bush and Dr. James Swain.

### SIXTY-THIRD LIST OF BOOKS.

The titles of books mentioned in previous lists are not repeated.

The figures in brackets refer to the figures after the names of the donors, and show by whom the volumes were presented. The books to which no such figures are attached have either been bought from the Library Fund or received through the *Journal.*

| | | |
|---|---|---|
| **Bérard, A. Poncet et L.** | *Traité clinique de l'Actinomycosis humaine* | 1898 |
| **Buxton, D. W.** | *Anæsthetics* .. .. .. .. .. .. .. 4th Ed. | 1907 |
| *Catalogue of Accessions to the Library of the Royal College of Physicians* | *of London* .. .. .. .. .. .. .. .. .. (5) | 1906 |
| *Catalogue of the Pathological Museum of the University of Manchester* | | 1906 |
| **Cathcart, C. W.** | *The Essential Similarity of Tumours* .. .. .. | 1907 |
| **Clubbe, C. P. B.** | *Intussusception* .. .. .. .. .. .. .. .. .. .. | 1907 |
| *Encyclopedia and Dictionary of Medicine and Surgery.* | Vol. III. .. | 1907 |
| **Haab, O.** .. | *Atlas of the External Diseases of the Eye* (Ed. by G. E. de Schweinitz) .. .. .. .. 2nd Ed. | 1906 |
| ,, .. | *Atlas and Epitome of Operative Opthalmology* (Ed. by G. E. de Schweinitz) .. .. .. .. .. | 1905 |
| **Hartmann, H.** | *Les Anastomoses intestinales* .. .. .. .. (1) | 1906 |
| **Hewitt, F. W.** | *Anæsthetics and their Administration* .. 3rd Ed. | 1907 |
| **Hurdon, H. A. Kelly and E.** | *The Vermiform Appendix and its Diseases* | 1905 |
| **Keetley, C. B.** | *The Prevention of Cancer* .. .. .. .. .. .. | 1907 |

**Kelly and E. Hurdon, H. A.** *The Vermiform Appendix and its Diseases* 1905
**Klein, E.** .. .. *The Bacteriology and Etiology of Oriental Plague* .. 1906
**McCaw, J.** .. *Aids to the Treatment of Diseases of Children.* 3rd Ed. 1907
**Moynihan, B. G. A.** *Abdominal Operations* .. .. .. 2nd Ed. 1906
*Needlandicorum, Opuscula Selecta. De Arte medica.* Fasc. I. .. 1907
**Owen, E.** .. *Cancer : its Treatment by Modern Methods* .. .. 1907
**Palmer, Margaret D.** *Lessons on Massage* .. .. .. .. 3rd Ed. 1907
**Poncet et L. Bérard, A.** *Traité clinique de l'Actinomycosis humaine* 1898
**Richardson, H.** *The Thyroid and Parathyroid Glands* .. .. .. 1905
**Ritchie, R. P.** *The Early Days of the Royal Colledge of Phisitians, Edinburgh* .. .. .. .. .. .. .. .. (1) 1899
**Savage, W. G.** *The Bacteriological Examination of Water Supplies* 1906
**Sutton, J. B.** .. *Tumours, Innocent and Malignant* .. .. 4th Ed. 1906
**Swanzy and L. Werner, H. R.** *Diseases of the Eye* .. .. 9th Ed. 1907
**Taves, A. W.** .. *The Etiology and Diagnosis of Epidemic Cerebro-Spinal Meningitis* .. .. .. .. .. .. .. .. 1906
**Wallace, F. C.** *Surgery of the Rectum* .. .. .. .. .. .. .. 1907
**Weber, Sir H. and F. P.** *Climatotherapy and Balneotherapy* .. .. 1907
**Werner, H. R. Swanzy and L.** *Diseases of the Eye* .. .. 9th Ed. 1907
**Whiteford, C. H.** *Glimpses of American Surgery in 1906* .. .. .. 1906
**Whiting, A.** .. *Aids to Medical Diagnosis* .. .. .. .. .. .. 1907

### TRANSACTIONS, REPORTS, JOURNALS, &c.

American Association of Obstetricians and Gynecologists, Transactions of the .. .. .. .. .. .. .. .. .. Vol. XVIII. 1906

American Dermatological Association, Transactions of the .. .. 1905

American Journal of Obstetrics, The .. .. .. .. Vol. LIV. 1906

American Ophthalmological Society, Transactions of the Vol. XI., Part 1 1906

Archives of the Middlesex Hospital .. .. .. .. (3) Vol. IX. 1906

Association of American Physicians, Transactions of the Vol. XXI. 1906

Boston Medical and Surgical Journal, The .. .. .. Vol. CLV. 1906

Bristol Directory, Wright's .. .. .. .. .. .. .. .. .. .. .. .. .. 1907

British Almanac, The .. .. .. .. .. .. .. .. .. .. .. .. .. 1907

British Medical Journal, The .. .. .. .. .. .. .. Vol. II. for 1906

Clinical Journal, The .. .. .. .. .. .. .. .. ..Vol. XXVIII. 1906

Edinburgh Medical Journal, The .. .. .. .. .. Vol. XX. 1906

Epidemiological Society of London, Transactions of the Vol. XXV. 1906

Gazette des Hôpitaux .. .. .. .. .. .. .. .. .. .. .. .. .. 1906

Gazette hebdomadaire des Sciences médicales de Bordeaux Tom. XXVII. 1906

Glasgow Medical Journal, The .. .. .. .. .. .. Vol. LXVI. 1906

Hazell's Annual .. .. .. .. .. .. .. .. .. .. .. .. .. .. 1907

Hospitals—

- Boston City Hospital, Medical and Surgical Reports of the .. (7) 1905

- Guy's Hospital Reports .. .. .. .. .. .. .. .. Vol. LX. 1906

- St. Bartholomew's Hospital Reports .. .. .. Vol. XLII. 1907

Journal of Tropical Medicine, The .. .. .. .. Vol. IX. 1909

Lancet, The .. .. .. .. .. .. .. .. .. .. .. Vol. II. for 1906

London County Council, Annual Report of the Medical Officer of the, for 1905 .. .. .. .. .. .. .. .. .. .. .. .. .. (2) 1906

Medical Chronicle, The .. .. .. .. .. 4th Series, Vol. XI. 1906

Medical Directory, The .. .. .. .. .. .. .. .. .. .. .. .. 1907

Medical Press and Circular, The .. .. .. .. [Vol. CXXXIII.] 1906

Medical Review, The .. .. .. .. .. .. .. .. .. Vol. IX. 1906

Montreal Medical Journal, The .. .. .. .. .. Vol. XXXV. 1906

Nord médical, Le .. .. .. .. .. .. .. .. .. .. .. .. .. 1906

Nordiskt Medicinskt Arkiv .. .. .. .. .. .. .. .. .. .. .. 1905

Odontological Society of Great Britain, Transactions of the Vol. XXXVIII. 1906

Ophthalmological Transactions .. .. .. .. .. Vol. XXVI. 1906

Otological Society of the United Kingdom, Transactions of the Vol. VII. 1906

Pathological Society of London, Transactions of the (4) Vols. LVI., LVII. 1905—1906

Practitioner, The .. .. .. .. .. .. .. .. .. [Vol. LXXVII.] 1906

Progrès médical, Le .. .. .. .. .. .. .. .. .. .. Tom. XXII. 1906

Progressive Medicine .. .. .. .. .. .. .. .. .. Vol. IV. 1906

Publishers' Circular, The .. .. .. .. (6) Vol. LXXXV. 1906

Revue hebdomadaire de Laryngologie .. .. .. .. Tom. XXVI. 1906

Sanitary Record, The .. .. .. .. .. .. .. .. Vol. XXXVIII. 1906

Scottish Medical and Surgical Journal, The .. Vol. XIX. 1906

Society for the Study of Disease in Children, Reports of the Vol. VI. 1905—1906

United States, Twelfth Census of the, 1900 .. .. (8) Vols. III., IV. 1902

West London Medical Journal .. .. .. .. .. Vol. XI. 1906

Whitaker's Almanack .. .. .. .. .. .. .. .. .. .. .. .. 1907

Year-Book of Scientific and Learned Societies, The .. .. .. .. 1906

Zentralblatt für Chirurgie .. .. .. .. .. .. .. .. .. .. .. 1906

Zentralblatt für Gynäkologie .. .. .. .. .. .. .. .. .. .. .. 1906

### MEETINGS OF SOCIETIES.

### Bristol Medico-Chirurgical Society.

### Local Medical Notes.

**University College, Bristol.**—A distinct step has been taken towards the University of Bristol by the complete incorporation of the Medical School with University College. The resolution agreeing to this was passed at a recent meeting of the Governors of the College. The present building has been used by the Medical Department for fourteen years, but there has so far been only co-operation between the two bodies. The present incorporation will increase the status of the School and College. The Medical School was at work more than forty years before the College was founded, and amongst its lecturers were some of the best men of the day. Hundreds of highly-trained men have been sent out by the School, which for so long stood in seclusion in the old Park—and Bristol medical men are to be found in all parts of the world.

**Long Fox Lecture.**—Dr. P. Watson Williams has been appointed lecturer for this year. It will be given in November, and the title and date will be announced in a later issue of this Journal.

**Examination Results.**—Students of the Medical Faculty have recently passed the following examinations:—

F.R.C.S. Eng.—*Primary Examination:* C. A. Joll, B.Sc. Lond.

Conjoint Board.—*Medicine:* P. S. Connellan, C. E. K. Herapath. *Surgery:* J. W. J. Willcox, J. Ellington Jones*, L.S.A. *Midwifery:* P. S. Tomlinson.

L.D.S., R.C.S. Eng.—*Mechanical Dentistry and Dental Metallurgy:* R. J Burton. *Final Examination, I.:* W. H. Ireland. *II.:* F. C. Nicholls*.

* Completes Examination.

**Bristol Royal Infirmary.**—C. F. Walters, F.R.C.S. Eng., has been appointed Surgical Registrar; W. W. King, M.R.C.S., L.R.C.P., Resident Obstetric Officer; and A. J. Turner, M.B., B.S. Durham, Junior House Surgeon.

**Bristol General Hospital.**—T. E. Coulson, M.B., Ch.B. Edin., has been appointed Senior House Surgeon.

The following description of the new Isolation Block at the Bristol General Hospital, opened by the Lord Mayor and Lady Mayoress of Bristol, in the unavoidable absence of Lady Smyth, who had promised to perform the ceremony, on January 26th, 1907, will be of interest.

The limited area of the site necessitated a somewhat unusual arrangement of plan. The building consists of two parts joined by a narrow, cross-ventilated neck. The south portion is devoted to accommodation for the patients and the northern part to that for nurses. The building contains two floors, the upper floor being reached by a separate staircase approached from the outside, so that there is no internal connection between the two floors. Each floor contains two wards of two beds each and one ward of one bed, with duty room, two bedrooms for nurses, and the usual baths and sanitary arrangements, stores, &c. Care has been taken to avoid crevices, where dust or other deleterious matter could be harboured ; all surfaces are smooth and easily cleaned. The doors are of the Gilmour type, now generally well known ; they have the appearance of being made out of a solid plank, but actually they are made up of a number of pieces, and veneered so that they present a perfectly smooth surface without ledges or sinkings ; they are finished with white enamel. The walls are plastered with cement and will ultimately be finished with enamel. The floors are of Euboeolith, a material laid in a plastic state, afterwards hardening and receiving polish. It is jointless, waterproof, fire-resisting, and unaffected by temperature. There is a heating chamber in the basement, and the warming is by hot water on the low-pressure principle, with ventilating radiators of a type easily cleaned. The extraction is by means of an electric fan.

At the Annual Meeting of the Bristol General Hospital, held on March 11th, under the presidency of the Lord Mayor, an excellent report for the past year was presented, which stated that exceptionally heavy demands had been made on the resources of the Hospital, both financially and in the work of treating a larger number of patients than has hitherto been recorded. The possession of the second operating theatre continued to lead to the increasing number of cases requiring operations of a more or less serious character being promptly dealt with. The isolation wing was now completed and fully equipped, and the formal opening recently took place. The work done at the Avonmouth Hospital under the medical officer and sister in charge had been considerable. The number of in-patients was 2,165, and that of the out-patients 35,264—a record number for the institution. 482 patients were sent to the convalescent home, with beneficial results. As regards finance, the ordinary expenditure exceeded the income—which had remained the same as in 1905—by £2,626. The workpeople's contributions amounted to £2,225, an amount which almost equalled the annual subscriptions. During the coming year it is hoped to raise £12,000, to enable two important additions to be made, which are needed to keep the institution thoroughly up to date. We feel sure that with the example of Bristol's public spirit quite fresh in our minds, which aided Sir George White to raise £50,000 for the Infirmary, this sum is not too much to ask for, considering the great work that is carried on at the General Hospital.

**Bristol Royal Hospital for Sick Children and Women.**—L. N. Morris, M.R.C.S., L.R.C.P., has been appointed Senior House Surgeon.

**Newport General Hospital.**—J. B. V. Watts, M.B., B.Sc. Lond., has been appointed House Surgeon.

**Bristol Dispensary.**—The committee of the Bristol Dispensary report that the number of patients recommended for relief in 1906 has been 10,323, viz. 7,997 at Castle Green and 2,326 at Bedminster, this being an increase over the previous year of 282. The number of notes sold to subscribers was 11,322. The committee much regret to record the death of their esteemed colleague, Mr. J. Hudson Smith, who had been a member of the committee for 22 years, and who generously remembered the institution in his will. W. H. A. Elliott, M.B., B.S.Lond., has been elected an additional surgeon to the institution.

**The Bristol Medical Dramatic Club,** which has now reached its twenty-ninth season, recently gave performances of *Home, Sweet Home, with Variations.* The performances were a great success, and the proceeds will be handed to the Medical Clubs' Union for the promotion of its endeavour to secure an athletic ground.

**Royal United Hospital, Bath.**—A public meeting was held at the Guildhall, Bath, on January 22nd, under the presidency of the Mayor, to consider the financial position of the Royal United Hospital, and the best means of reducing the debt of £5,000 which exists on the institution. The Mayor will make an effort to wipe off the debt during his year of office, and stated that he had already received several subscriptions for this purpose. After some discussion the meeting pledged itself to support the Mayor in his admirable determination to place the Royal United Hospital on a sound financial basis.

**South Devon and East Cornwall Hospital, Plymouth.**—Lionel Shingleton Smith, M.R.C.S., L.R.C.P., has been appointed House Surgeon to this hospital.

**Devon and Cornwall Ear and Throat Hospital.**—The twentieth annual meeting of the subscribers to the Devon and Cornwall Ear and Throat Hospital, Plymouth, was held on February 5th, under the presidency of the Mayor. The medical report stated that during 1906 there had been 987 patients treated, compared with 859 in 1905, and that 23 in-patients had been admitted against 12 in the previous year.

" **Index Medicus.**"—An intimation has been issued by the Carnegie Institution of Washington that " unless it appears that the *Index Medicus* is of greater service to the medical profession, and can help to support itself to a greater extent than in the past, it may become advisable to discontinue its publication." The *Index Medicus* was established in 1879, and died for want of support in 1899. In 1903 the Carnegie Institution made a grant of £2,000 a year to keep it alive, whilst the subscription price was reduced from £5 to 25s. per annum. We understand that the total number of subscribers is 532, of whom 396 are in the United States. All those engaged in bibliographical research will remember how difficult and tedious searching for references became when the *Index Medicus* ceased in 1899, and it is to be hoped that many doctors—we will not say medical libraries, as we hope there are none who are not subscribers—will acknowledge the generosity of the Carnegie Institution by becoming subscribers. The subscription (25s.) defrays only about one-fifth of the cost of production.

**Pathological Demonstrations.**—On February 14th Dr. J. M. H. Eyre, of Guys Hospital, London, gave a demonstration on Pneumococci at the Bristol Eye Hospital, upon the invitation of Professor Walker Hall. Dr. Eyre pointed out the differences in the several types of pneumococci, and deprecated the dependence upon morphological characters alone for routine diagnosis. He insisted that numerous cultural tests should be carried out before any diplococcus was designated as the pneumococcus. He also remarked upon the manner in which the tissues resisted the pneumococcal organisms. In some cases a cellular, in others a fibrinous exudation, marked the response. Dr. Eyre considers that pneumonia is a distinct septicæmic condition, the cocci being present in the blood stream at an early period of the disease.

On February 18th Professor Walker Hall gave a lantern demonstration on epidemic cerebro-spinal meningitis and malaria and blood diseases at the Royal Infirmary. He showed some of the new serum, detailed its mode of preparation, and explained the manner of its application.

We understand that Professor Symmers, of Belfast, is shortly expected to take one of these demonstrations. The subject will be "Bilharziosis." Professor Symmers was able to obtain a large number of specimens during his tenure of office in Cairo. Bilharziosis is now becoming so common in this district, that we feel sure everyone will be glad of an opportunity of discussing the condition.

We are enabled to state that the coming summer "Friday" demonstrations will assume rather a different character from those of the past winter session. Dr. Walker Hall proposes to devote the entire term to the diseases of the stomach and intestines. The bacteriology, parasitology, and chemistry of the tract will be considered in detail, and the ulcerations and obstructions will be dealt with fully. A feature of the course will be the condition of the gastric and intestinal mucosæ and contents in diseases of the circulatory, respiratory, renal, and nervous systems. Practitioners will be admitted on presentation of their cards.

